# Treatment of Acute Pneumonia

**Published:** 1893-01-21

**Authors:** 


### GLAMORGANSHIRE AND MONMOUTHSHIRE INFIRMARY, CARDIFF.

### Treatment of Acute Pneumonia.

The treatment of acute pneumonia forms one of the most instructive chapters in the history of medicine, and illustrates in a striking way the vicissitudes and fortunes of the healing art during the last half-century. When the present consulting physicians, fresh from the influence of the Paris school, joined the staff of the Cardiff Infirmary, bleeding was in full force, and resorted to almost in every case as a matter of routine, in much the same way as poultices are used at the present day. Depressing drugs, such as antimony, in the form of tartar emetic and James' powder, assisted, in the views of that time, to "crush out" the disease. The inevitable reaction came, and the restorative treatment, with its large quantities of brandy, had its day. Since then various drugs have appeared on the scene, each claiming to shorten the course or lessen the mortality of the disease. Among them quinine, digitalis, aconite, veratria, and the alkalis were the most prominent, but even these have been abandoned by their friends, except in combating special symptoms. All this violent swinging of the pendulum has not been without its value, for it has taught the broad principles upon which a disease, like pneumonia, naturally tending to cure, should be treated. Belief in "systems" is now dead, and it is recognised that the only rational treatment is to carefully consider the value of the various symptoms as they arise, the extent to which they affect the patient's comfort and recovery, and accordingly require treatment or not. Many circumstances have to be carefully weighed, and above all it is important to remember that the patient, rather than the disease, is the primary object of treatment.

Of the local applications used at the Cardiff Infirmary in the treatment of acute pneumonia, perhaps the majority of cases have on admission linseed-meal poultices. These are applied to the affected side and renewed every three hours. This affords early relief to the pleuritic pain frequently so prominent, and of late years have been entirely limited to this end. A feeling that continuous hot poulticing is very exhausting to the patient, without much corresponding benefit, has led to the adoption of a simple cotton wool jacket, which retains the heat sufficiently, and, moreover, avoids the necessity of disturbing the patient. It may be that the success of cold applications to the chest, introduced of recent years, has drawn attention to this point, as it is certainly the case that routine poulticing is experiencing the fate of other "systems," and falling rapidly into disuse. For severe pain hot fomentations, with or without a few drops of turpentine, are used, as counter-irritation can be more rapidly carried out. Blisters are, as a rule, avoided, on account of the discomfort and inconvenience of a tender, raw surface. When there is much distress with the pains leeching is resorted to, three to six being placed over the spot where pain is located; if necessary, hot fomentations are applied subsequently to encourage bleeding.

To relieve the cough and promote expectoration, such remedies as the expectorant medicines, ipecacuanha wine, compound tincture of camphor, and squills in the form of tincture, syrup, or hymel are given. In sthenic cases, antimonial wine has been long a favourite for the same purpose, serving, as it does, further to moderate the rapidity of the circulation and stimulate the skin. In children, the bronchitis kettle with a tent bed is occasionally used, but, like poulticing, not to the extent it was formerly.

For the promotion of sleep, one of the chief points in the treatment, the different hypnotic remedies are in use. When pain is present, its relief is important. When the insomnia is not due to pain, small doses of the bromides and chloral hydrate, or in form of bromidia, are given frequently; and of the newer drugs, sulphonal and chloralamide have proved valuable. A combination of tincture of digitalis and chloral hydrate, in the proportion of seven drops of the former to five grains of the latter, given every four hours—as recommended by Dr. Balfour, of Edinburgh—is sometimes employed, and found to act well in cases advanced in life, where there is much restlessness with weak heart action. Alcohol, in the form of brandy, is also used as a hypnotic, half an ounce to one ounce being given at bed-time.

In severe dyspnœa, with a tendency to cyanosis, venæsection has been resorted to on a few occasions within the last ten years, with relief certainly, though of a temporary character. Each time not more than ten or twelve ounces of blood were withdrawn. Recently the employment of oxygen inhalations has been tried, the apparatus being that supplied by the Brins Oxygen Company. Much good result has not so far been obtained, but the treatment has not been sufficiently tried to determine with accuracy the limits of its uses.

To control pyrexia, quinine has always been a favourite drug, in doses varying from five to twenty grains, as often as the circumstances require. Aconite is used by others, who believe it of great value for this purpose, in drop doses of the tincture every quarter of an hour for the first hour, and then every hour afterwards until the temperature falls. Salicylate of soda is likewise employed, but more rarely on account of its tendency to produce unpleasant symptoms. Of the newer anti-pyretics, antipyrin in doses of ten grains every two or three hours, and antifebrin in smaller quantities are as yet used sparingly. On the whole, it is only in a few that antipyretic drugs have been found of any value. Cold, in the form of baths, cold effusions to the chest, and the cold pack, have not been tried. Recently, however, at the Union Hospital Dr. Sheen has used the ice-bag in over twenty cases of pneumonia, all of which recovered. It was found to give much relief to the symptoms, and in a few there was reason to think that the spread of the disease was arrested and its duration shortened. On several occasions, it was striking to observe how a patient, semi-delirious with severe pain, became calmed and fell asleep after the ice-bag had been on the affected side half an hour. It always had a favourable influence on the temperature, and the further advantage that the effect could be readily controlled. This, of course, places it, as an antipyretic agent, above the usual drugs, the notorious uncertainty of which has been the subject of much scoffing at the hands of cynics, from Voltaire downwards. The ice-bag is the most convenient form of applying cold, and if care be taken to have a tight-fitting cap which does not leak, the patient may be kept perfectly dry and comfortable. It is kept on the chest until the temperature falls below 101 deg., and reapplied on any rebound.

*Delirium*, when it is marked, is generally treated by stimulants, brandy being the form used. In Dr. Sheen's cases the ice-bag had a very decided effect in relieving this symptom. In alcoholic patients with delirium the bromides with chloral are mostly relied upon.

Where the acute stage is past, and the lung resolving but slowly, counter-irritation by iodine or small fly blisters is had recourse to, while iodide of potash and cinchona mixture is administered internally, and, later on, tonics containing iron, quinine, strychnine, &c.

